# Oral lesions and immune status of HIV infected adults from eastern Nepal

**DOI:** 10.4317/jced.50888

**Published:** 2013-02-01

**Authors:** Giridhar S. Naidu, Rachana Thakur, Asutosh K. Singh, Srijana Rajbhandary, Rajeev K. Mishra, Alok Sagtani

**Affiliations:** 1 (Former) Assistant Professor, Department of Oral Medicine and Radiology. College of Dental Surgery, B.P.Koirala Institute of Health Sciences, Dharan, Sunsari District, Nepal; 2 Dental Surgeon. College of Dental Surgery, B.P.Koirala Institute of Health Sciences, Dharan, Sunsari District, Nepal; 3 Associate Professor, Dept of Oral and Maxillofacial Surgery. College of Dental Surgery, B.P.Koirala Institute of Health Sciences, Dharan, Sunsari District, Nepal

## Abstract

Objective: To document the prevalence, age and gender distribution of oral lesions in HIV infected adults and the influence of highly active antiretroviral therapy and correlate them to the immune status of the patients. Materials and Methods: Oral lesions were diagnosed by a detailed physical examination by trained and calibrated examiners according to the case definitions established by the Oral HIV/AIDS research alliance. Demographic details, risk behavior patterns and oral symptoms and habits were collected by a questionnaire. Results: 81 patients; 54 men and 27 women aged between 20 – 55 years participated in the study. A total of 49 patients; 60.5% had some oral lesion when examined. Oral candidiasis (21 %) and oral melanosis (21%) were the most common lesions, followed by linear gingival erythema, oral hairy leukoplakia, necrotizing ulcerative periodontitis/gingivitis, herpes labialis, parotid gland enlargement and reccurent apthous ulcers. Oral hairy leukoplakia was exclusively seen in men (p=0.018). All six cases of herpes simplex lesion were seen in non - anti retro viral group (p=0.073) while oral candidiasis was commonly noted in the anti retro viral group (p=0.073). Lowering CD4 counts had the strongest association with the prevalence of oral candidasis (p=0.012), pseudomembranous candidiasis (p=0.014) and oral hairy leukoplakia (p= 0.065). Conclusion: This study shows a high prevalence of oral candidiasis, melanosis, linear gingival erythema and oral hairy leukoplakia in the patients.

** Key words:**HIV, AIDS, oral lesions, prevalence.

## Introduction

Acquired Immunodeficiency Syndrome (AIDS) has emerged as a global crisis since its discovery in 1981 with the epicenter now firmly located in Asia and Africa (1). Nepal, home to some 23 million people has more than a hundred ethnic and linguistic groups (2). A decade long armed conflict here has had a tremendous repercussion on the overall development in the country adding to poverty, deprivation and discrimination. In this period, the Human Immunodeficiency Virus (HIV) status has evolved from a “low” to a “concentrated” epidemic (3) after the first case of AIDS here was reported in 1988. As of 2011, national estimates indicate that approximately 50,200 adults and children are infected with HIV with a probable overall prevalence of about 0.30% in the adult population (15-49 years old)(4). The epidemic in Nepal is driven by injecting drug use and heterosexual transmission (5). The highest burden of the epidemic is shared by the highway districts (Terai region) where nearly 50% people with HIV are living (4). The scarcity of reports on the oral manifestations of HIV from the eastern region of the highway districts (Eastern Terai) helped conceive the present study. In this context, we studied the clinical presentations, prevalence, age & gender distribution of oral lesions and the influence of antiretro viral (ARV) therapy in HIV sero-positive adult patients in eastern Nepal and correlated these parameters to their immune status.

## Material and Methods

A total of 81 adult patients with HIV infection/AIDS comprised the sample for the cross-sectional study. The patients were either attending the out patient clinic of the departments of Tropical diseases and General medicine or were directly recruited from 6 HIV rehabilitation centres in eastern Nepal. The study was conducted during a period of 5 months, from June 2008 to October 2008. The study had the approval of the research committee of the institution and the informed consent of the participating patients’.

HIV serology of all patients was performed at the central laboratory of our institution and included the enzyme-linked immunosorbent assays (HIV Tridot Test, J.Mitra Co, India and HIV ELISA, Merind Diagnostics, Belgium). Two separate positive tests were considered confirmatory in all cases. All patients were evaluated for CD4 T- lymphocyte counts [Fluorescence-Activated Cell Sorter Count (FACS) machine BD Biosciences FACS count SW 1.5/4/05] after staining patient’s blood with monoclonal antibodies. Patients were examined within one month of obtaining the CD4 lymphocyte counts. A short questionnaire was used to collect data on demographic details, risk behavior patterns and oral deleterious habits. Details of drugs taken were retrieved from the medical records of the patients.

The physical examination was carried out by a specialist in Oral Medicine and either of two dental surgeons. The examiners were trained and calibrated on ten HIV/AIDS patients with multiple oral lesions. The examiners were neither aware of the HIV clinical stage and the CD4 cell count nor whether the patient was on anti-retro viral (ARV) therapy or not. A standard oral examination recommended by World Health Organization (WHO) was followed under artificial light. The extra oral and peri-oral tissues were examined first followed by intra oral tissues for changes in size, color and shape of anatomical areas as well as for clinical signs and lesions. The oral lesions associated with HIV infection were diagnosed based on case definitions established by the Oral HIV/AIDS research alliance (6) and photographic records were made of the lesions.

## Statistical Analysis

The data obtained were encoded, entered and analyzed using SPSS version 11.5. Patients were categorized by gender, clinical stage, age and ARV therapy. Prevalence of oral lesions in different groups and comparisons between groups of total sample were performed using the Chi Squared test. In situations where 5% of cells had expected count less than 5 Fisher’s exact test was used. Bivariate analysis by Spearman Rho’s rank correlations was used to find the association between CD4 counts and occurrence of lesions, influence of ARV therapy. A p-value of <0.05 was considered significant. Inter-observer variability between examiners was assessed by Kappa statistic.

## Results

The study included 54 men and 27 women (M: F ratio of 2:1) with a combined mean age for men and women at 32.493 years. The women were younger at a mean of 30.25 years than the men at 33 years. Majority of the patients 67(82.7%) were in the age group 20-39 years. The distribution of CD4 count between various groups is summarized in  Table 1. The average CD4 counts were more in the women 379.70cells/cu mm than the men 252.51 cells /cu mm.

**Table 1:**
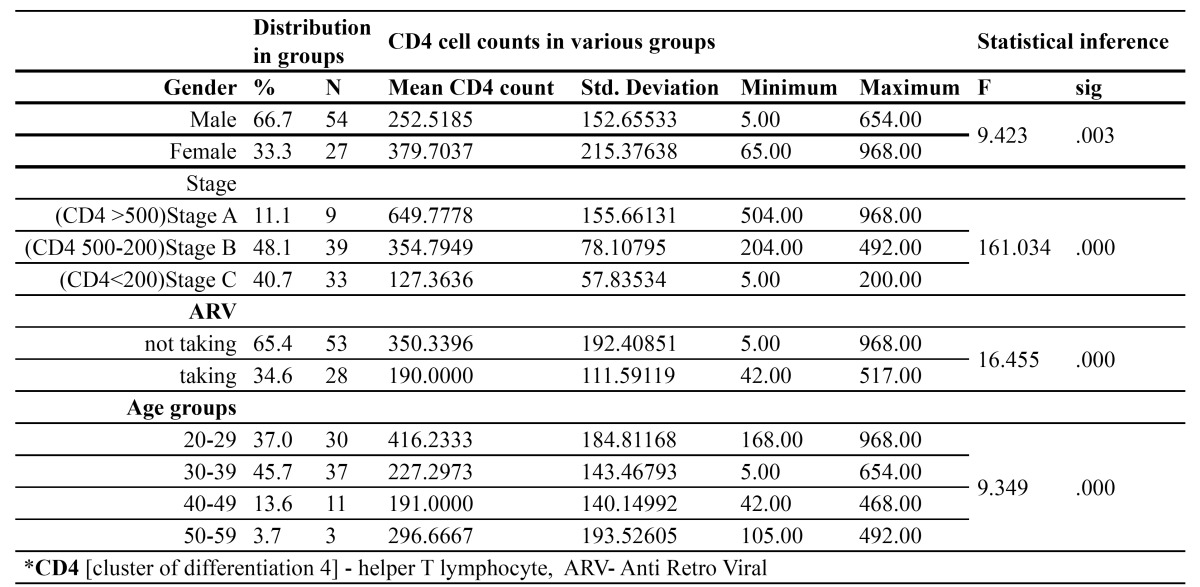
Showing the distribution of CD4* cell counts in gender, ARV therapy, disease stage & age groups.

**Figure 1:**
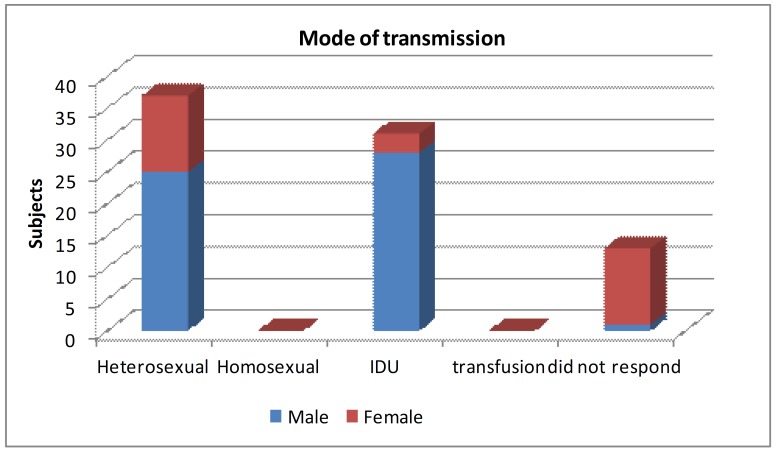
Showing Distribution of patients based on mode of transmission of HIV infection.

**Figure 2:**
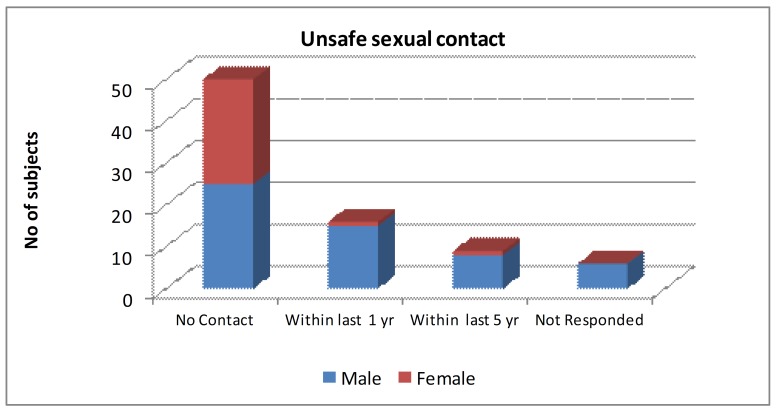
Showing distribution of patients based on unsafe sexual contact history preceding HIV infection.

Mode of transmission is summarized in Fig. 1 and Fig. 2. About 31 patients; 38.3% indulged in injected drug use (IDU) and 25 patients; 30.9% had unsafe sexual contact with commercial sex workers within the past 5 years.

Oral lesions of HIV/AIDS were seen in 49 patients; 60.5% who included 32 men and 17 women. The number of oral lesions ranged from single to a maximum of 4 per subject. The most common diagnosis was oral candidiasis [OC] (21 %) and oral melanosis[OM] (21%) followed by linear gingival erythema[LGE] (17.3%), oral hairy leukoplakia[OHL] (12.3%), necrotizing ulcerative periodontitis/ginigvitis[NUP/NUG] (8.6%), herpes labialis (7.4%), parotid gland enlargement [PGE] (3.7%) and reccurent apthous ulcers (1.2%). Other lesions observed but not included in the HIV case definitions were leukoplakia (13 cases; 16%), tobacco pouch keratosis (10 cases; 12.3%) and lichenoid lesions (3 cases; 3.7%). The inter-observer agreement in the identification of oral lesions of HIV/AIDS was found to be 0.85 by Kappa statistics.

 Table 2 shows distribution of oral lesions of HIV/AIDS by gender and ARV therapy. Thirty-two men had oral lesions as compared to 17 women. All 10 cases of OHL were seen exclusively in men (p=0.018). Fifty three patients; 65.4% had not received any anti retro viral therapy. Oral lesions of HIV /AIDS were seen in 19 pa-tients; 67.8% on ARV therapy as opposed to 44 patients; 83% not on ARV therapy. The most common oral lesion in patients’ on highly active anti retro viral therapy [HAART] was oral candidiasis (9 cases;32.1 %) and oral melanosis (9 cases;32.1%) followed by linear gingival erythema [LGE], oral hairy leukoplakia [OHL], erythematous candidiasis [EC], pseudomembranous candidiasis[PC] and necrotizing ulcerative periodonti-tis/ginigvitis [NUP/NUG]. In the patients’ not on HAART, erythematous candidiasis (9 cases; 17%) was seen frequently followed by LGE, herpes labialis and OHL. All six cases of herpes simplex lesion were seen in non - anti retro viral group (p=0.073) while oral candidiasis was commonly noted in the anti retro viral group (p=0.073).

**Table 2:**
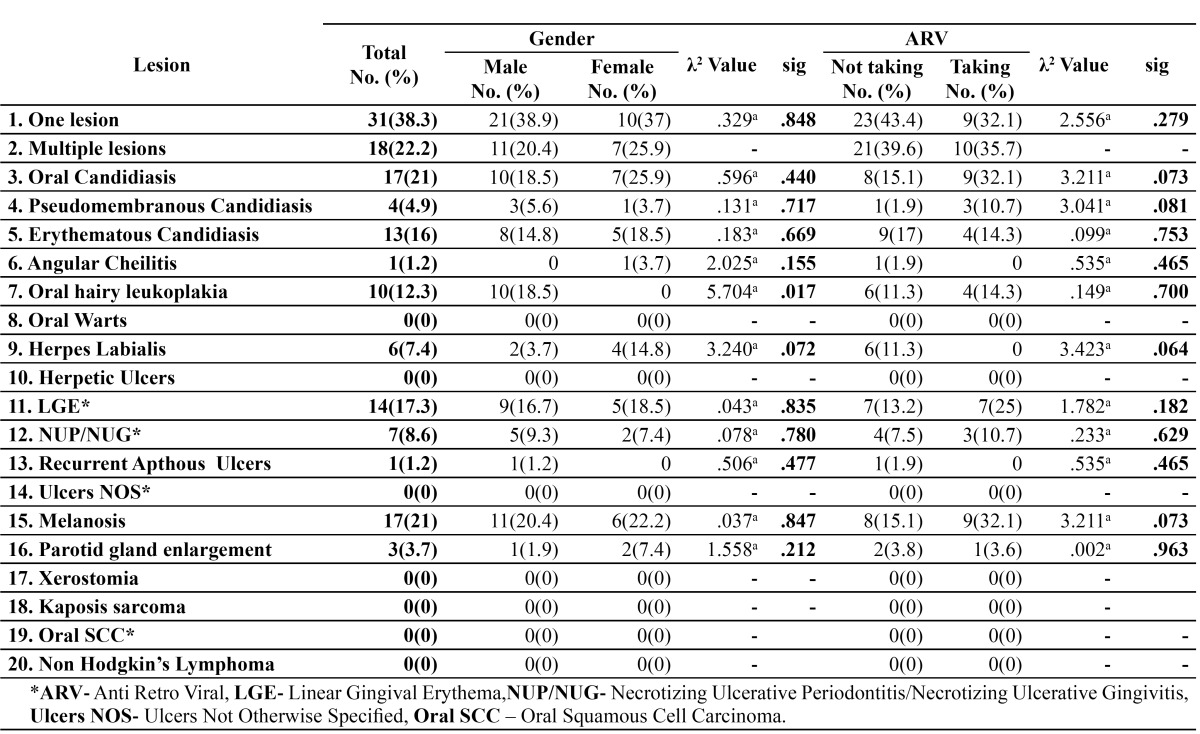
Distribution of lesions by gender and ARV* therapy.

Distribution of oral lesions by stage of disease along with associations with CD4 counts are summarized in  Table 3. Pseudomembranous candidiasis was found to be exclusive to stage C disease with CD4 counts < 200 cells/cu mm(p= 0.47). Associations between falling CD4 counts and the increased prevalence of oral lesions was found to be strongest for OC (p=0.012), PC (p=0.014) and OHL (p= 0.065).

**Table 3:**
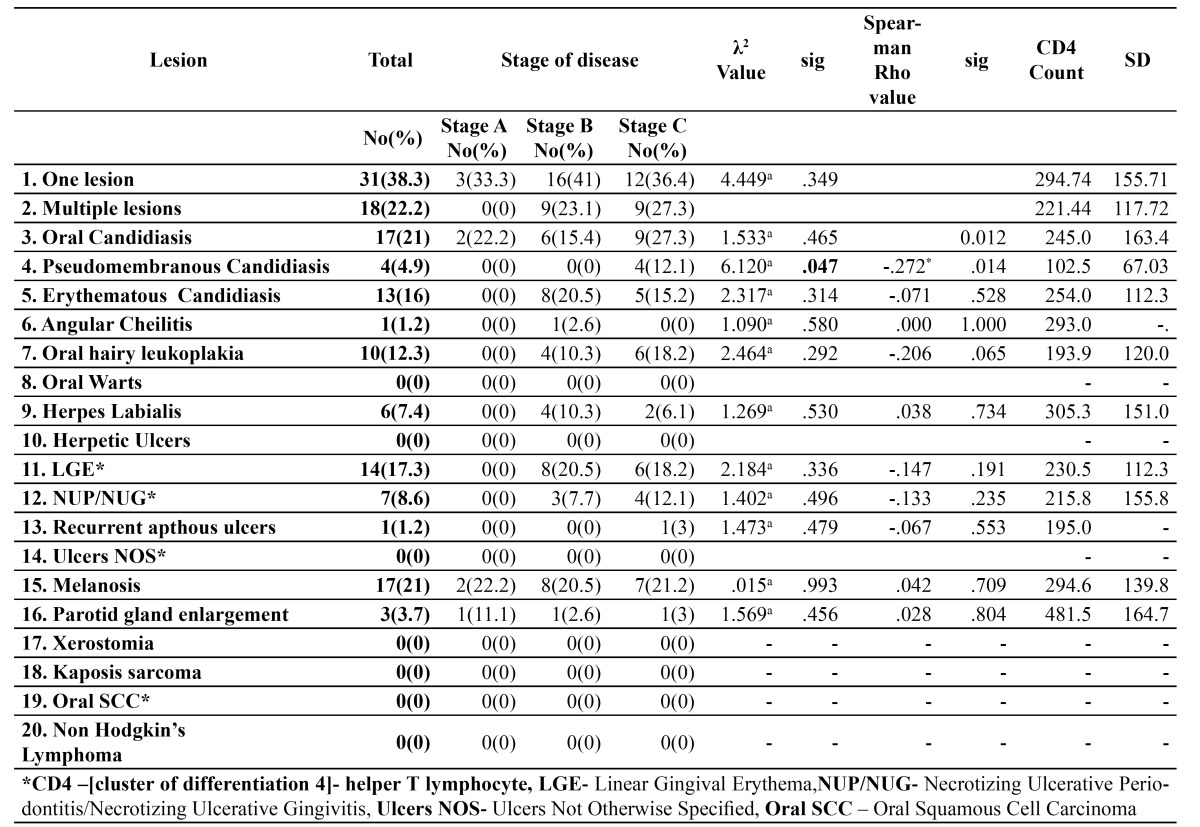
Distribution of lesions by disease stage and association with CD 4 cell counts.

## Discussion

Nepal’s recent history replete with political strife, armed conflict and poor development has allowed its progression from a “low” to a “concentrated” HIV epidemic status after the first case of AIDS here was reported in 1988. The paucity of data on oral lesions in HIV infected adults from the Asian subcontinent and the multi ethnic nature of Nepal’s population makes comparison a challenge. Patients in our study were frequently men 54 cases; 66.7% with an M: F ration of 2:1 as compared to the national average of 2.9:1.The average age of 32.49 years was similar to other studies from the subcontinent (1). The peak age distribution of 20-39 years reflects the national data on HIV prevalence in Nepal. The largest number of HIV positive cases 42% was reported among men and women aged 30 to 39 years followed by 22% of 24 to 29 year olds. The anticipated decrease in working force could thereby have an adverse effect on the socioeconomic status of the country.

Heterosexual contact in 37 cases; 45.7% closely followed by injected drug use in 31 cases; 38.3% was the major route of transmission. This trend was seen in most Asian studies except the Thailand study (7) where intravenous drug users constituted the majority. Although men having sex with men (MSM) constitute 4% of the HIV infected patients from the national data (4) we did not find any such cases.

Oral lesions of HIV/AIDS were seen in (49 cases; 60.5%) patients. However, previous studies in Thailand (82 %) (8), India (86%) (9) and Cambodia (90%)(10)have reported a higher prevalence. The lesions ranged from a single to a maximum of 4 lesions per subject. CD4 counts appeared to decrease with age (-0.403,p=0.000). Pseudomembranous candidiasis was found to be exclusive to stage C disease with CD4 counts < 200 cells/cu mm(p= 0.47).The frequency of oral lesions increased with lowering CD4 counts and the strongest association was with the prevalence of OC (p=0.012), PC (p=0.014) and OHL (p= 0.065).

Oral candidiasis , the most common lesion in our study (21%), was lower than previously recorded in India 50.4%(1), 56%(9), 82%(11), 81%(12) and 32.25%(13). However, other Asian studies from Thailand 24.8 %(8), Cambodia 22.8% (10) and Singapore 35%(14) showed similar prevalence. Of the oral candidiasis, erythematous candidiasis (13 cases; 16%) was the most common followed by pseudomembranous candidiasis (4 cases; 4.9%) and least by angular cheilitis (1 case; 1.2%). Since a majority of our participants were in stage B clinical disease (mean CD4 count of 354.7 cells/cu mm) and the patients in stage C disease were receiving ARV therapy the above trends could be expected. It was interesting to note that PC was noted only in patients with CD4 count < 200 cells/cu mm (p= 0.47). EC predominated in the stage B and stage C of clinical disease. No forms of candidiasis were detected in stage A clinical disease patients, clearly affirming the fact that candidiasis is a proven clinical marker for advanced immuno-suppression.

Oral melanosis (17 cases;21%) was found to be the other most common lesion presenting as patchy brown areas over the labial, buccal and palatal mucosa. Nine patients; 32.1 % on ARV presented with melanosis which was double in percentage terms, than the non-ARV group (8 cases; 15.1%). Mixed prevalence has been reported from India of about 30% (13). Melanotic hyperpigmentation is well documented to be a side effect of HAART therapy (1). However, we found no statistically significant difference in the distribution based on ARV therapy (p=0.73). Furthermore, tobacco smoking can cause grayish pigmentation of the oral mucosa, but showed a poor association with the patchy brown pigmentation in our study(r=0.043, p=0.704). This suggests that ARV therapy and tobacco smoking may not be directly responsible for this peculiar pigmentation seen in HIV infection advocating the need for investigation in further studies.

Oral hairy leukoplakia seen in 10 patients (12.3%) was comparable to findings of other Asian studies 13% (8), 13% (15) , 12%(16), and 11%(17), but slightly more than reported in Indian studies 3%(9), 2.1%(11), 7%(12), 4.13%(13).

Fourteen cases; 17.3% of linear gingival erythema was the other common finding in a higher percentage of women (5 cases; 18.5%) as observed by Sharma et al. (25%) (1). Necrotizing ulcerative gingivitis/periodontitis (7 cases; 8.6%) was seen less frequently when compared to studies from Cambodia 28 % (15) and Thailand 15 %(18) but, more than reported from India 5.64 %(13). The recent inclusion of NUP and NUG as a single group in the revised case definitions has implications in the prevalence previously reported.

Ulcerative lesions in HIV infection can be due to recurrent apthous stomatitis (RAS), herpes simplex (HSV), cyto-megalo virus(CMV), varicella zoster virus(VZV) infections and necrotizing ulcerative stomatitis (NUS), while all other ulcerative lesions are recorded as ulcers not otherwise specified (NOS). Six cases; 7.4% with herpes simplex lesions presenting as herpes labialis were recorded and treated with topical 5% acyclovir therapy. While oral herpes zoster lesions were absent; we however, examined a case of cutaneous herpes zoster of the chest. One case of recurrent apthous ulcers was noted in a male patient with CD4 count of 195cells/cu mm. Ulcerative lesions were an uncommon finding in our study. Since laboratory detection was not employed definitive diagnosis of the above mentioned ulcerative lesions was not possible. Hence, it may well be an incorrect estimation of the true prevalence.

Salivary gland disease (SGD) in HIV/AIDS includes xerostomia and enlargement of salivary glands. We found three cases; 3.7% of bilateral parotid gland enlargement which was more than the 1% reported from Thailand (18). Persistent circulating and visceral CD8 lymphocytic infiltration of parotid glands described as Diffuse Infiltrative Lymphocytic Syndrome (DILS) has been proposed to be the reason for the salivary gland enlarge-ment in HIV/AIDS(20). Further, xerostomia has been shown to occur in patients with HAART (21), due to an antisecretory action on acinar cells. In our study all the three cases of salivary gland disease (SGD) manifesting as parotid enlargements were in patients not receiving ARV therapy while xerostomia was not recorded in any patient. Xerostomia has been reported from India at a prevalence of 16.12 %(13).

Kaposi’s sarcoma has not been reported in other Asian studies. This has been explained due to the endemic absence of Human herpes Virus -8 in populations of these regions (21). Our sample size was insufficient to come across cases of non-Hodgkin’s Lymphoma (NHL) which usually has a low average prevalence of 3-5%. Oral squamous cell carcinoma was similarly not recorded in any of the patients of the study.

ARV therapy was made available to 28 patients who received highly active anti retro viral therapy (HAART), comprising either two Nucleoside Reverse Transcriptase Inhibitors (NRTI) + one Protease inhibitor (PI) or two NRTI + one Non-Nucleoside Reverse Transcriptase Inhibitors (NNRTI). As a policy, HIV seropositive patients were monitored through regular checkups and instituted antibacterials, antifungals and vitamins. When patients showed rapid disease progression or were symptomatic with CD4 counts falling below 200 cells/cu mm HAART was initiated and maintained till CD4 counts stabilized. The true effects of HAART could not be estimated since treatment was initiated recently in a majority of patients. The majority of patients who were on HAART had been receiving treatment for a period of less than 6 months. Thereby, the lower average CD4 counts (190 cells/cu ml, SD 111.59) in the ARV group could be expected as a consequence. Recent guidelines of threshold CD4 counts for the initiation of HAART have been revised from 200 to 350 cells/cu ml in mid and low-income countries (22). This may lead to a substantial increase in the number of patients who will be treated with HAART and the consequential change in the prevalence of oral lesions. The most common lesions in the ARV group were oral melanosis (9 cases; 32.1 %) and oral candidiasis, (9 cases; 32.1 %). In the patients not on ARV therapy erythematous candidiasis, (9 cases; 17%) was frequently seen followed by melanosis, (8 cases; 15.1 %). All six cases; 7.4% of herpes simplex lesion were seen in non - anti retro viral group (p=0.073) while oral candidiasis was commonly noted in the anti retro viral group (p=0.073).

Other oral lesions can occur due to deleterious habits in patients with HIV/AIDS; hence we further investigated tobacco and alcohol habits and their association to oral lesions. Leukoplakia (11 cases; 20.4%) and tobacco pouch keratoses (9 cases; 16.7%) were seen more frequently in men. Tobacco consumption as khaini, gutka or cigarette was strongly associated with prevalence of leukoplakia (r=0.238, p=0.33) and tobacco pouch keratoses (r=0.265, p=0.017). Lichenoid lesions (3 cases; 3.7%) showed poor association with tobacco habits (r=0.139, p=.217). None of the oral lesions of HIV/AIDS showed a strong association with tobacco and alcohol habits.

This study is unique since the above findings haven’t yet been documented from this population. The present study shows a high prevalence of oral candidiasis, oral melanosis , LGE and OHL in the HIV infected patients. As in other parts of the world understanding the nature of the disease and its manifestations will lead to invol-vement of all sections of the healthcare system including the dental profession which may largely have remained isolated here.

The revised National Policy on HIV and STI, 2011 has highlighted a preventive approach for the National HIV/AIDS Strategy for 2011-2016. Encouragingly, a significant drop in HIV prevalence among injected drug users from 68% (2003 estimates) to 6.3 % (2011 estimates) has been observed. (5)

The national antiretroviral treatment programme has also received a boost as until December 2011, 23.7 per cent (6,051 adults, 432 children) were accessing ARV therapy from 26 ARV treatment sites and 10 sub-ARV treatment sites throughout the country, a substantial expansion of ARV therapy sites from two at the start of 2004 (5). The role of the dental profession cannot be overstated in pitching in by early HIV/AIDS case identi-fication and making a credible contribution to fighting this dreaded disease.
